# The activity of the serotonergic 5-HT_1A_ receptor is modulated by voltage and sodium levels

**DOI:** 10.1016/j.jbc.2022.101978

**Published:** 2022-04-22

**Authors:** Merav Tauber, Yair Ben Chaim

**Affiliations:** Department of Natural and Life Sciences, The Open University of Israel, Ra'anana, Israel

**Keywords:** G protein–coupled receptors, 5-HT receptors, voltage dependence, Xenopus oocytes, sodium dependence, cRNA, circular RNA, DR, dose response, GIRK, G protein–activated inward-rectifying K^+^, GPCR, G protein–coupled receptor, 5-HT, 5-hydroxytryptamine, I_5-HT_, 5-HT-induced K^+^ current, M2R, M2 muscarinic receptor

## Abstract

G protein–coupled receptors are known to play a key role in many cellular signal transduction processes, including those mediating serotonergic signaling in the nervous system. Several factors have been shown to regulate the activity of these receptors, including membrane potential and the concentration of sodium ions. Whether voltage and sodium regulate the activity of serotonergic receptors is unknown. Here, we used Xenopus oocytes as an expression system to examine the effects of voltage and of sodium ions on the potency of one subtype of serotonin (5-hydroxytryptamine [5-HT]) receptor, the 5-HT_1A_ receptor. We found that the potency of 5-HT in activating the receptor is voltage dependent and that it is higher at resting potential than under depolarized conditions. Furthermore, we found that removal of extracellular Na^+^ resulted in a decrease of 5-HT potency toward the 5-HT_1A_ receptor and that a conserved aspartate in transmembrane domain 2 is crucial for this effect. Our results suggest that this allosteric effect of Na^+^ does not underlie the voltage dependence of this receptor. We propose that the characterization of modulatory factors that regulate this receptor may contribute to our future understanding of various physiological functions mediated by serotonergic transmission.

G protein–coupled receptors (GPCRs), the largest protein family in the body, are of great physiological and pharmacological importance. Binding of an external agonist promotes coupling of the GPCR to its cognate G protein, and this, in turn, induces downstream signaling. Several factors have been proposed to regulate the affinity and activity of these receptors, including allosteric modulators (1), arrestins (2), and receptor activity–modifying proteins (3).

In recent years, membrane potential has emerged as a new surprising modulator of GPCR activity. Several studies, employing different approaches, revealed that membrane potential can modulate the affinity and activity of many GPCRs, including receptors for acetylcholine (4, 5), glutamate (6), dopamine (7, 8, 9), adrenaline (10, 11), purines (12, 13), opioids (14), and prostanoids (15). The molecular mechanism that underlies the effect of membrane potential on GPCRs is not yet fully understood. For muscarinic receptors, it has been suggested that the orthosteric binding site of the M2 muscarinic receptor (M2R) (*i.e.*, the site that binds ligands that can lead to the activation of the receptor) is involved in the voltage dependence. Evidence suggests that this receptor undergoes depolarization-induced conformational change, which underlies the change in the affinity of the receptor (16). Specifically, a conserved tyrosine lid above the orthosteric binding site (17) was proposed to serve as a voltage-sensing motif in the M2R. It was further suggested that the allosteric binding site is involved in this process as well (16, 18). Furthermore, for this receptor, research postulates that the G protein–coupling site may also have a role in determining the voltage dependence (19, 20). In addition, it is quite well established that Na^+^ has an allosteric effect on family A GPCRs. Studies suggest that Na^+^ modulates the affinity and activity of several GPCRs, including dopaminergic, adrenergic, and adenosine receptors (21, 22, 23) (reviewed in Ref. (24)). Data regarding several GPCRs established the structural basis for Na^+^ effect on GPCRs and highlighted a conserved aspartic acid in position 2.50 to be crucial for this effect (22, 24, 25). More recently, a link between the modulatory effects of membrane potential and Na^+^ has been suggested based on modeling approaches (26, 27), predicting movement of Na^+^ from its binding site upon changes in membrane potential. Experimental evidence for the existence of such a link is still lacking.

Serotonin (5-hydroxytryptamine [5-HT]) is a neurotransmitter involved in many physiological functions, in both the central nervous system and the periphery (28, 29, 30). 5-HT exerts its effect by activating a family of receptors comprised of seven subfamilies (28). All 5-HT receptors, except the 5-HT_3_ receptor (which forms a ligand-gated ion channel), are GPCRs. 5-HT receptors mediate numerous signal transduction processes (31, 32) and have been implicated in many pathologies, including depression and anxiety (29, 33). In this study, we focused on the 5-HT_1A_ receptor. Binding of 5-HT (or other agonists) to the 5-HT_1A_ receptor leads to activation of G_i/o_-protein mediated signaling, which normally causes cell hyperpolarization and an inhibition of action potential firing (34). The 5-HT_1A_ receptor is highly expressed on serotonergic neurons in the dorsal and median raphe nuclei, where it functions as a presynaptic autoreceptor that modulates the release of 5-HT (35). 5-HT_1A_ receptors are also expressed postsynaptically in many brain regions including the hippocampus and cortex (36). Although the 5-HT_1A_ receptor has been studied extensively, its voltage and Na^+^ dependences have not yet been investigated. In the current study, we investigate these modulatory effects using Xenopus oocytes as an expression system.

## Results

To study the voltage dependence of the 5-HT_1A_ receptor, Xenopus oocytes were injected with circular RNAs (cRNA)s of proteins involved in the pathway leading to activation of K^+^ currents by the receptor *via* βγ subunits of the G-proteins: 5-HT_1A_ receptor, the two subunits of the G protein–activated inward-rectifying K^+^ (GIRK) channel (GIRK1 and GIRK2), and the Gα_i3_ subunit (4, 37).

We first verified that 5-HT does not exert a receptor-independent effect on the GIRK channels. To do so, we measured the effect of 5-HT on oocytes expressing the GIRK channel but not the receptor. Fig. S1 shows that 5-HT does not affect the GIRK channel directly, thus enabling us to use the 5-HT-induced GIRK currents as a measure for receptor activation.

**Figure 1:**
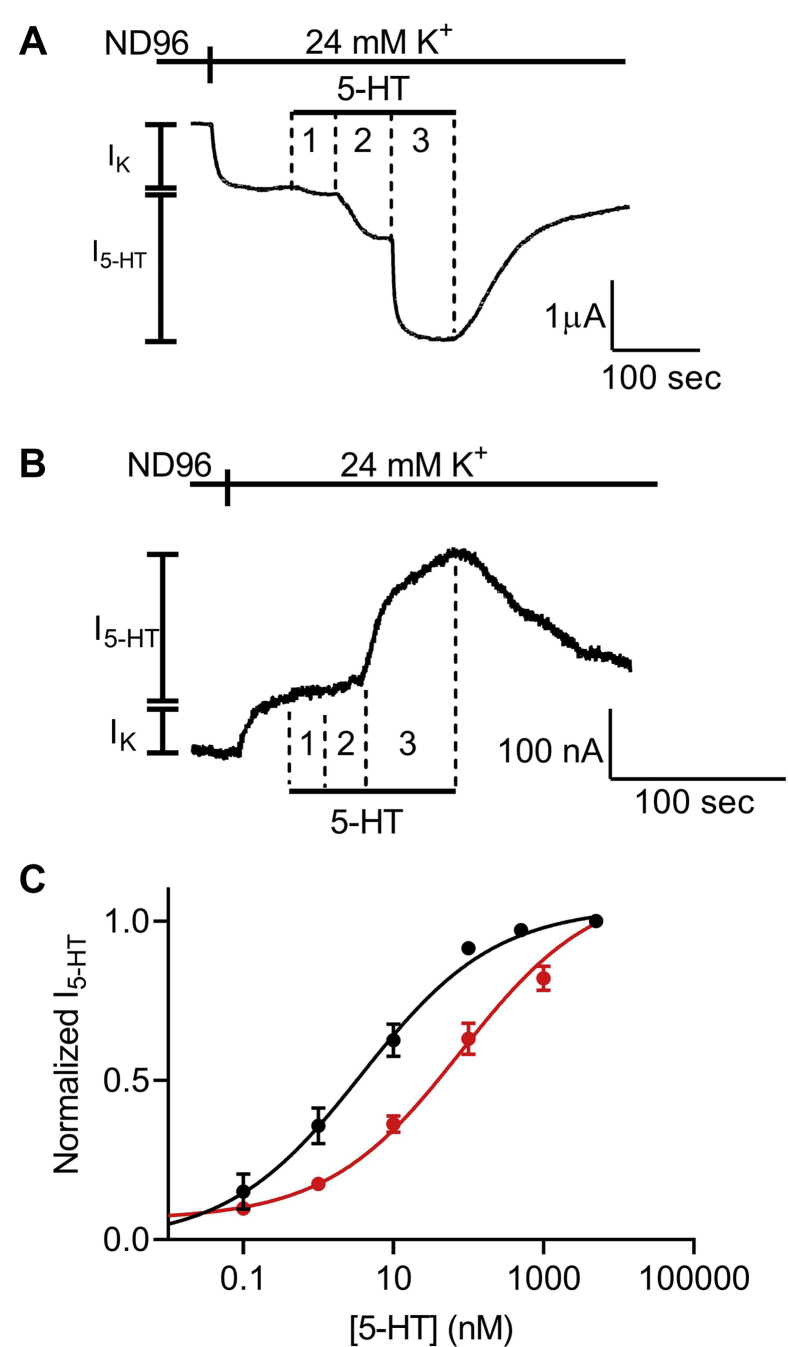
**Voltage dependence of the 5-HT**_**1A**_**receptor.***A* and *B*, measurement of the relationship between 5-HT concentration and 5-HT_1A_ receptor–activated GIRK currents at −80 mV and +40 mV, respectively. Basal GIRK current evolved following replacement of the solution to a high K^+^ solution. Then, three different 5-HT concentrations were applied (1, 10, and 5000 nM, numbered 1–3), and the response for each concentration was measured. *C*, dose–response curves for −80 mV (*black*) and +40 mV (*red*). The responses were normalized to the response evoked by 5000 nM 5-HT at each holding potential. Each point represents the mean (±SEM) from 11 to 50 oocytes. The *solid black and red lines* were generated by fitting equation 1 to the data (see the Experimental procedures section). The EC_50_ values obtained for the two graphs were significantly different (*p* < 0.0001). 5-HT, 5-hydroxytryptamine; G protein–activated inward-rectifying K^+^.

Next, the dependence of the 5-HT-induced K^+^ current (I_5-HT_) on 5-HT concentration (dose response [DR]) was measured at two holding potentials: –80 mV and +40 mV. Figure 1, *A* and *B* depicts the basic experimental protocol for three 5-HT concentrations. The oocyte was voltage clamped to either –80 mV (Fig. 1*A*) or +40 mV (Fig. 1*B*), in a low K^+^ (2 mM K+) solution, ND96 (see the Experimental procedures section). Basal GIRK current (I_K_) was developed upon replacement of the ND96 by the 24 mM K^+^ solution. Then, three concentrations of 5-HT were applied sequentially, giving rise to I_5-HT_. I_5-HT_ was terminated upon washout of 5-HT. Employing this basic experimental protocol, full DR curves at the two holding potentials were constructed. For each holding potential, I_5-HT_ (*i.e.*, the current evoked by 5-HT above the basal I_K_ at any particular 5-HT concentration) was normalized to I_5-HT_ obtained at a saturating concentration of 5-HT (5 μM; higher 5-HT concentration did not evoke higher GIRK currents) at the same holding potential. This enabled us to compensate for the intrinsically different GIRK currents obtained at the two holding potentials of –80 mV and +40 mV in a single oocyte and to compare between oocytes. Figure 1*C* depicts the cumulative results from eight batches of oocytes. The results are both from oocytes where data were obtained from one of the holding potentials and recordings where the same oocyte was subjected to both holding potentials. The results of the latter (shown in Fig. S2) were not different from the cumulative results. The results suggest that membrane potential affects the apparent affinity of 5-HT toward the 5-HT_1A_ receptor. Specifically, depolarization decreases the potency of this ligand in activating the 5-HT_1A_ receptor by more than 20-fold. The EC_50_ was 3.8 nM at −80 mV and 85.3 nM at +40 mV.

**Figure 2:**
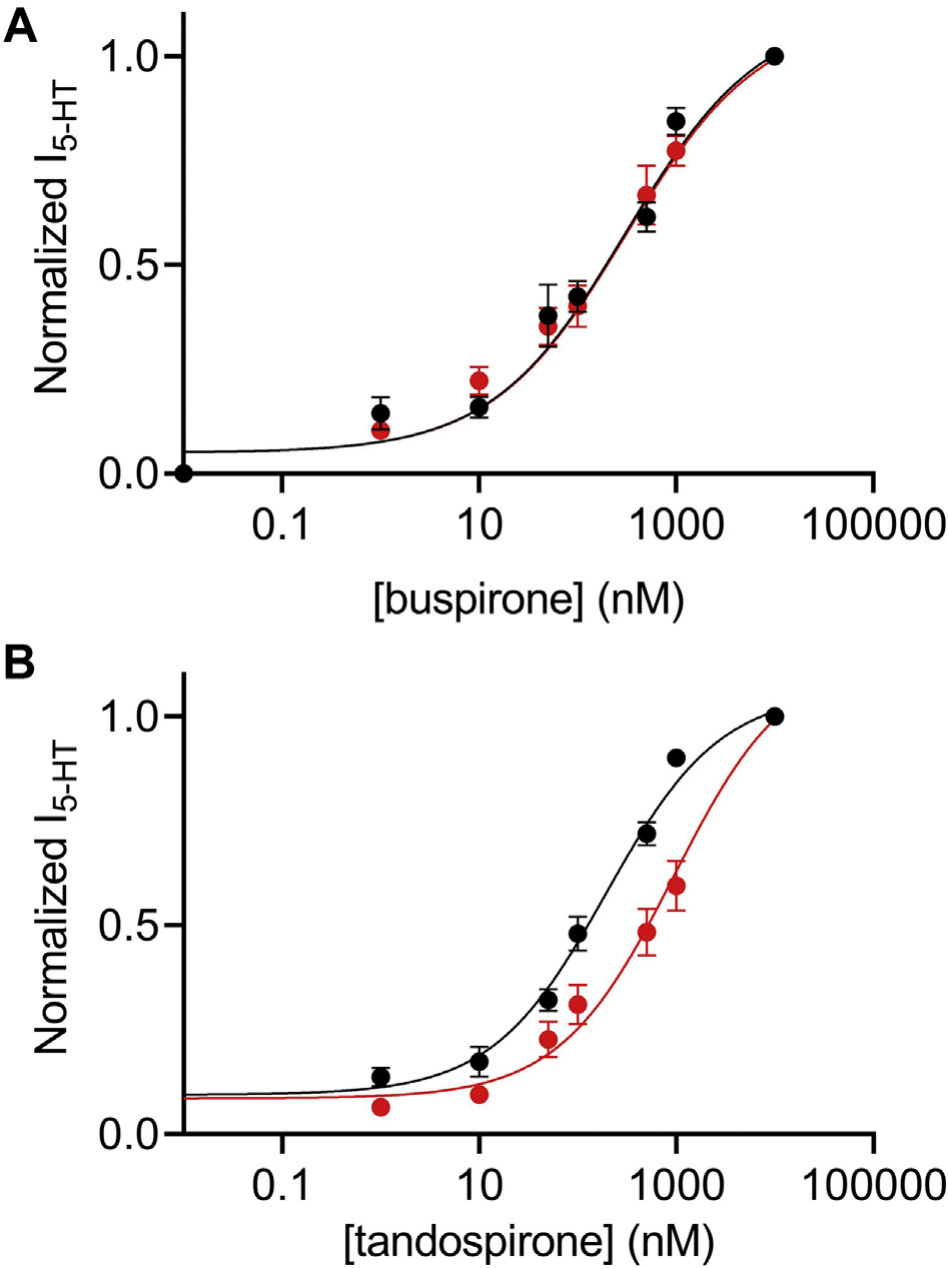
**Voltage dependence of the activation of the 5-HT**_**1A**_**receptor by buspirone and tandospirone.***A*, dose–response curves for the activation of the 5-HT_1A_ receptor by buspirone at −80 mV (*black*) and +40 mV (*red*). Each point here and in *B* represents the mean (±SEM) from 12 to 32 oocytes. The *solid black* and *red lines* were generated by fitting Equation 1 to the data (see the Experimental procedures section). The EC_50_ values obtained for the two graphs (301.9 nM at −80 mV and 313.7 at +40 mV) were significantly different (*p* < 0.0001). *B*, dose–response curves of tandospirone activated 5-HT_1A_ receptor. The EC_50_ values obtained for the two graphs (194 nM at −80 mV and 934.1 nM at +40 mV) were significantly different (*p* < 0.0001). 5-HT, 5-hydroxytryptamine.

Voltage dependence of GPCRs has been shown to be agonist dependent. Namely, while the potency of one agonist may decrease upon depolarization, the potency of others may not be voltage dependent or even increase when the membrane potential is depolarized (5, 8). The 5-HT_1A_ receptor is a target for a variety of therapeutically relevant drugs. As the potency of these drugs may be affected by membrane potential, we sought to investigate the voltage dependence of the activation of this receptor by two agonists that are used pharmaceutically. We first used buspirone, which is an agonist to the 5-HT_1A_ receptor that is widely used as an anxiolytic drug (38, 39). This ligand was reported to act either as a full agonist or as a partial agonist for the 5-HT_1A_ receptor (40, 41). In our experiential system, buspirone acts as a partial agonist; the maximal response evoked by buspirone (10 μM) was ∼42% of the maximal response produced by 5-HT. Similar maximal activation was observed at both holding potentials, suggesting that voltage does not affect the efficacy of this ligand in activating the receptor (Fig. S3*A*). The DR curves, obtained under two membrane potentials as described previously, are depicted in Figure 2*A*. In contrast to the activation by 5-HT, the potency of buspirone was not voltage dependent. The EC_50_ values obtained for the two membrane potentials were not significantly different (277.8 nM at −80 mV and 313.7 at +40 mV). Similarly, we examined the voltage dependence of another anxiolytic drug, tandospirone. As reported before (42, 43), we found that tandospirone acts as a partial agonist; it evokes a maximal response that is ∼55% of the maximal response evoked by 5-HT at the same oocytes. The maximal response was similar at both holding potentials, suggesting that voltage does not affect the efficacy of this agonist in activating the receptor (Fig. S3*B*). The DR curves obtained using this ligand are depicted in Figure 2*B*. Our results suggest that this ligand activates the receptor in a voltage-dependent manner, although its voltage dependence was weaker than that of 5-HT. The EC_50_ value at −80 mV (194 nM) was approximately five times lower than the one obtained at +40 mV (937.9 nM).

A known allosteric modulator of class A GPCRs is Na^+^ ions (24). For several family A GPCRs, it was shown that Na^+^ allosterically regulates the binding of agonists. Furthermore, theoretical studies have proposed that movement of Na^+^ from its binding site may be the source of the charge movement–associated currents in these receptors and thereby may underlie their voltage dependence (26, 27).

Experimental evidence regarding the effect of Na^+^ on the 5-HT_1A_ receptor function is still lacking, to the best of our knowledge. Thus, to investigate whether extracellular Na^+^ ([Na^+^]_o_) underlies the voltage dependence of the activation of the 5-HT_1A_ receptor by 5-HT, we first explored whether Na^+^ modifies 5-HT_1A_ receptor activation. To this end, we repeated the experiments described previously in Na^+^-free solution (Na^+^ was replaced by the large ion *N*-methyl-d-glucamine; see the Experimental procedures section; to ensure full removal of Na^+^ from the extracellular solution, the oocyte was bathed in Na^+^-free solution for at least 5 min prior to the recording). We have previously shown that removal of extracellular Na^+^ does not affect the GIRK currents themselves (44), and therefore, receptor-activated GIRK currents may be used in order to study the effect of Na^+^ on 5-HT_1A_ receptor activation.

**Figure 3:**
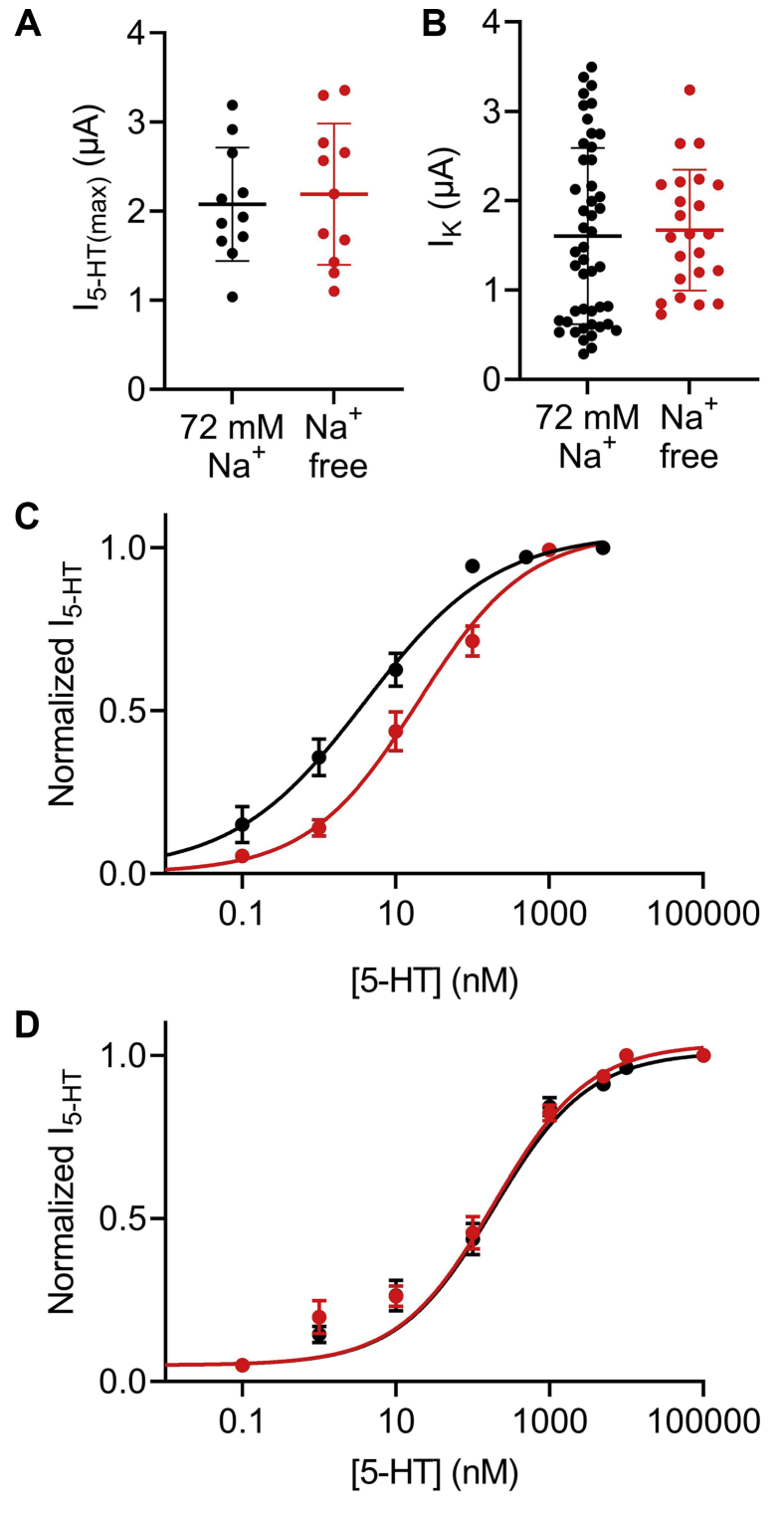
**Allosteric effect of sodium ions on the 5-HT**_**1A**_**receptor.***A*, the maximal amplitude of I_5-HT_, evoked by 5 μM 5-HT in 72 mM Na^+^ solution (*black*) and in Na^+^-free solution (*red*). Each data point represents one oocyte, and the mean (±SD) is shown as a *horizontal line*. The data at the two conditions are not significantly different (unpaired *t* test, *p* = 0.79). *B*, basal I_K_ from experiments in 72 mM Na^+^ solution (*black*) described for Figure 1 and in Na^+^-free solution (*red*). Each data point represents one oocyte, and the mean (±SD) is shown as *horizontal lines*. The data at the two conditions are not significantly different (unpaired *t* test, *p =* 0.76). *C* and *D*, DR curves assembled from various experiments conducted on wt 5-HT_1A_ receptor (*C*) or Asp92Asn mutant (*D*) at 72 mM Na^+^ solution (*black circles*) and in Na^+^-free solution (*red circles*). Each point represents the mean (±SEM) from 8 to 31 oocytes. The *solid black* and *red lines* were generated as described previously for Figure 1. The EC_50_ values obtained for the two graphs at *C* are significantly different (*p =* 0.016). The EC_50_ values obtained for the two graphs at *D* are not significantly different (*p =* 0.15). 5-HT, 5-hydroxytryptamine; DR, dose response; I_5-HT_, 5-HT-induced K^+^ current.

The results of these experiments are depicted in Figure 3. As illustrated, the removal of Na^+^ from the extracellular solution did not affect the maximal current of the receptor in our experimental system (Fig. 3*A*). Furthermore, the basal GIRK current measured from the oocytes that were used in this experiment did not differ from the basal currents measured in the experiments described in Figure 1, indicating that the expression levels were similar at both groups of oocytes (Fig. 3*B*). Constructing DR curves revealed an allosteric effect of Na^+^ on the potency of 5-HT in activating the 5-HT_1A_ receptor. Comparing the DR curve obtained under these conditions with the DR curve obtained at 72 mM Na^+^ (Fig. 3*C*), we found that removal of Na^+^ affected the potency of 5-HT in activating the receptor. Specifically, 5-HT showed lower potency in activating the 5-HT_1A_ receptor at Na^+^-free solution (*red*) in comparison to the potency at 72 mM Na^+^ (*black*; the EC_50_ was 23.03 nM at Na^+^-free conditions, in comparison to 3.8 nM in 72 mM Na^+^, taken from the data of Fig. 1*C*). It is interesting to note that while this observation is in line with our recent finding concerning the M2R, where the removal of Na^+^ decreased the affinity of acetylcholine toward the receptor (44), this observation is not consistent with results from other GPCRs, where the removal of Na^+^ increases the affinity of the receptor (22, 23).

Several structural studies suggested that Na^+^ acts *via* binding at a specific binding site within the helical bundle. These and other functional studies implicated a conserved aspartate residue in transmembrane domain II (Asp2.50) as being involved in the allosteric effect of Na^+^ on class A GPCRs. Mutating this residue to an uncharged residue results in the abolishment of the Na^+^ dependence in other GPCRs (45, 46, 47). To validate the role of this residue, Asp82 in the 5-HT_1A_ receptor, in Na^+^ binding, we mutated it to asparagine (Asp82Asn) and repeated the experiments described previously. Figure 3*D* depicts the results, demonstrating that while the potency of the Asp82Asn mutant was lower than that of the wildtype 5-HT_1A_ receptor, the Na^+^ dependence of the potency of the ligand was diminished. The two EC_50_ values (163.9 nM in 72 mM Na^+^ and 226.5 nM in Na^+^-free solution) were not significantly different. These results suggest that Asp 2.50 indeed plays a role in the allosteric effect of Na^+^ on 5-HT_1A_ receptor function.

**Figure 4:**
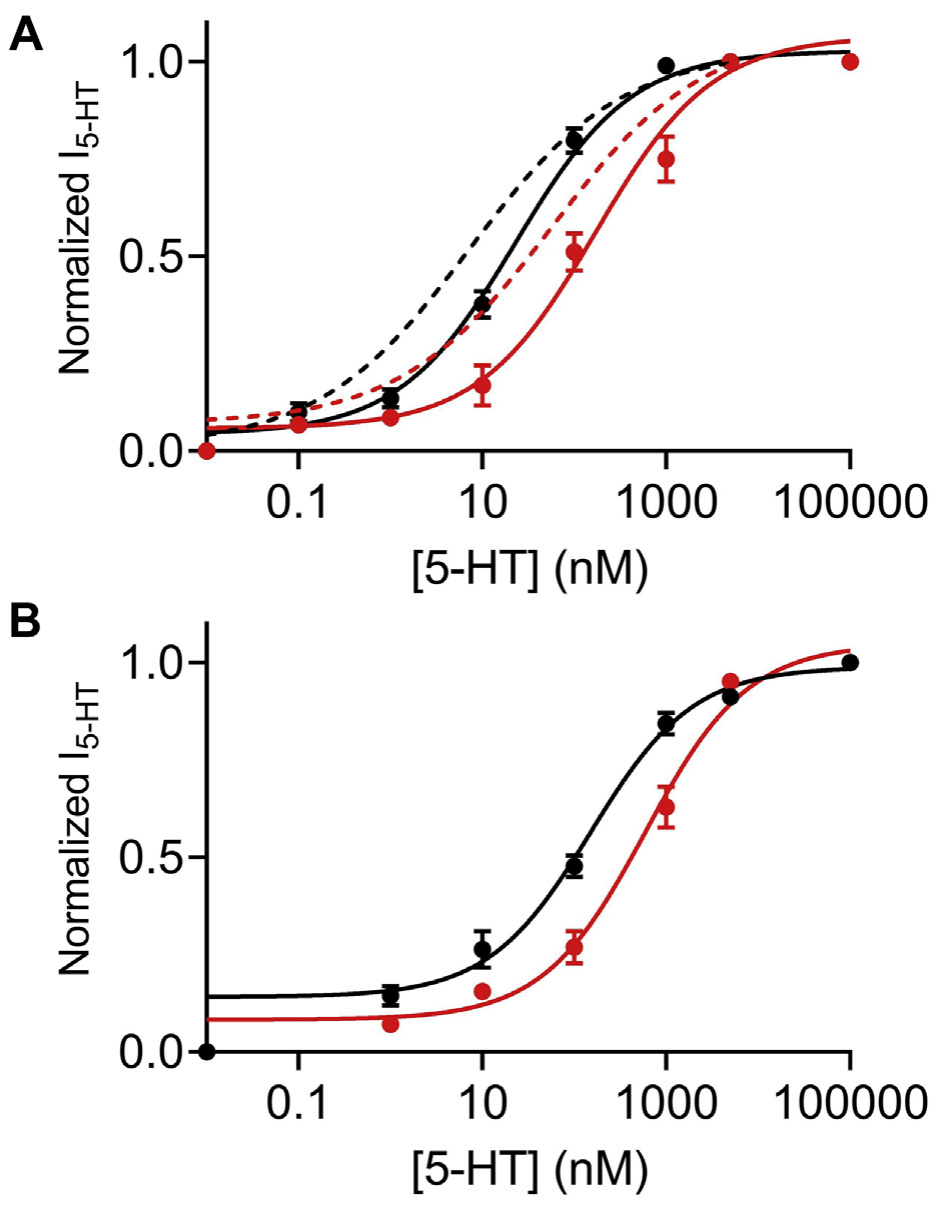
**Effect of sodium ions on the voltage dependence of the 5-HT**_**1A**_**receptor.***A*, dose–response curves assembled from various experiments conducted on wt 5-HT_1A_ receptor in Na^+^-free solution at −80 mV (*black circles*; taken from Fig. 2*B*) and at +40 mV (*red circles*). Each point represents the mean (±SEM) from 7 to 21 oocytes. The *solid black* and *red lines* here and in *B* were generated as described for Figure 1. The *dashed lines* are the fitting curves of the data obtained in 72 mM Na^+^ (taken from Fig. 1). The EC_50_ values obtained for the two graphs (19.4 nM at −80 mV, taken from Figure 3*A*, and 170.4 nM at +40 mV) are significantly different (*p =* 0.0018). *B*, dose–response curves of Asp82Asn mutant receptor in 72 mM Na^+^ solution at −80 mV (*black circles*; taken from Fig. 3*C*) and at +40 mV (*red circles*). Each point represents the mean (±SEM) from 8 to 31 oocytes. The EC_50_ values obtained for the two graphs (126 nM at −80 mV and 595.1 nM at +40 mV) are significantly different (*p =* 0.0018). 5-HT, 5-hydroxytryptamine.

To further investigate whether the allosteric effect of Na^+^ indeed underlies the voltage dependence of the 5-HT_1A_ receptor, we measured the potency of 5-HT in activating the 5-HT_1A_ receptor in Na^+^-free solution at +40 mV as well. The results (Fig. 4*A*) indicate that the removal of Na^+^ did not abolish the voltage dependence of 5-HT potency (*black and red symbols* and *solid line* represent data obtained at Na^+^-free solution at −80 mV and +40 mV, respectively; the *dashed black and red lines* represent the data obtained at the respective membrane potentials at 72 mM Na^+^). However, it did affect the potency in a voltage-dependent manner. Namely, while it had a pronounced effect on DR curve obtained at −80 mV (approximately sixfold increase in EC_50_ value, see aforementioned), it had a less robust effect on the potency of 5-HT at +40 mV (EC_50_ = 170.9 nM, less than twofold decrease in EC_50_ in comparison to the EC_50_ obtained at 72 mM Na^+^). These results suggest that the voltage dependence of 5-HT potency toward the 5-HT_1A_ receptor is not determined solely by Na^+^ sensitivity of this receptor. To further test this conclusion, we examined the effect of the Asp82Asn mutant on the voltage dependence of the receptor. Previous studies concerning the M2R showed that although this mutation diminished the charge movement–associated currents in this receptor (5), it did not abolish its voltage dependence (17, 48). The results for the 5-HT_1A_ Asp82Asn mutant (Fig. 4*B*) were consistent with the results obtained with the M2R. Namely, the mutation in Asp82 did not abolish the voltage dependence of the 5-HT_1A_ receptor. The EC_50_ values obtained for the two membrane potentials were significantly different (163.9 nM at −80 mV and 595.1 at +40 mV). Taken together, the results of Figure 4 suggest that the allosteric effect of Na^+^ on the 5-HT_1A_ does not serve as the main mechanism by which voltage modulates the receptor's activity.

## Discussion

GPCRs play a critical role in many signal transduction processes in the body relating to health and disease. Thus, it is not surprising that the allosteric mechanisms that regulate these receptors have been studied extensively. In family A GPCRs, such mechanisms may include the binding of allosteric ligands (49) or sodium ions (24, 50) to the receptor, which in turn modulate its affinity and activity. Another allosteric modulator of GPCRs is the membrane potential, which was shown to affect the affinity of several GPCRs (51). It has been proposed that these allosteric mechanisms may be linked to each other. A study that was conducted on muscarinic receptor subtypes suggested that the allosteric site regulates the voltage sensitivity of these receptors (18). Other studies utilized a molecular dynamics modeling approach to suggest that the voltage dependence is linked to the binding and unbinding of sodium ions to the receptor (26, 27).

The current study focuses on the 5-HT_1A_ receptor. We found that the potency of 5-HT in activating this receptor is higher at resting potential (−80 mV) than under depolarization (+40 mV). Interestingly, we found that this voltage dependence is agonist specific. While the potency of the 5-HT_1A_ receptor agonist tandospirone was voltage dependent, although its voltage dependence was weaker than that of 5-HT, the potency of buspirone was shown to be voltage independent. This agonist specificity was observed also for other GPCRs (5, 8) and may have implications in the design of new drugs that differ from the endogenous ligands. We further established that Na^+^ is able to modulate the potency of 5-HT toward the 5-HT_1A_ receptor. Removing Na^+^ from the extracellular solution lowered the potency of 5-HT in activating the receptor. Finally, we found that Na^+^ is probably not the main factor that governs the voltage dependence of this receptor. The receptor exhibited voltage dependence even in the virtual absence of extracellular Na^+^.

The effect of membrane potential on biological molecules has been investigated for several decades. Most studies focused on voltage-gated ion channels, where the voltage-sensing motif has been identified and characterized (52). Much less is known regarding the voltage dependence of GPCRs. Since these proteins do not contain a motif that resembles the canonical voltage sensor of ion channels, other mechanisms have been examined for their role in voltage dependence of GPCRs. This includes the implications of several sites at the vicinity of the ligand binding or at the G protein–coupling site as involving in the voltage dependence. Our results further suggest that Na^+^ does not play a crucial role in the voltage dependence of the 5-HT_1A_ receptor, although the allosteric effect of Na^+^ appears to be somewhat voltage dependent. The removal of Na^+^ had a more pronounced effect at −80 mV than at +40 mV. This may indicate that the two allosteric mechanisms share some structural features. It is possible to speculate that depolarization shifts the receptor into a low-affinity state, which resembles the low-affinity conformation that predominates in the absence of Na^+^. Thus, the effect of these two factors may not be additive. Further investigation of the interplay between different allosteric mechanisms is required in order to elucidate the mechanism that governs the affinity of the receptor under such conditions.

The physiological importance of voltage dependence of GPCRs is starting to unfold (53, 54). A well-studied example is the process of neurotransmitter release, where it was proposed that voltage-induced conformational change in the receptor decreased the affinity of the receptor and thereby weakens the interaction of the receptor with the release machinery, and consequently induces transmitter release (55). Direct evidence to support this chain of events has recently been demonstrated for cholinergic synapses (56). 5-HT_1A_ receptors play a similar inhibitory role as presynaptic autoreceptors that regulate 5-HT release in the dorsal raphe nuclei (57). Therefore, it is possible that a similar mechanism is present in these synapses, as well. Given the importance of the 5-HT_1A_ receptor in many physiological functions (58, 59), a further exploration of this receptor in a more physiological setting has the potential to enhance our understanding of the molecular mechanisms that underlie these pathological states.

## Experimental procedures

### Ethics statement

All experimental procedures used in this study were performed in accordance with relevant guidelines and regulations and approved by the Hebrew University’s Animal Care and Use Committee (ethical approval number: NS-11-12909-3).

### Preparation of cRNA and oocytes

cDNA plasmids of the two subunits of the GIRK (GIRK1 and GIRK2), the 5-HT_1A_ receptor (kindly provided by Dr Erhard Wischmeyer from the University of Wurzburg, Germany) (60), and the α subunit of the G-protein (Gαi3) were linearized with the appropriate restriction enzymes (44). The linearized plasmids were transcribed *in vitro* using a standard procedure. Point-directed mutagenesis was done using Quick-Change II Site-Directed Mutagenesis Kit (Stratagene).

*Xenopus laevis* oocytes were isolated and incubated in NDE96 solution composed of ND96 (in millimolar: 96 NaCl, 2 KCl, one CaCl_2_, one MgCl_2_, 5 Hepes, with pH adjusted to 7.5 with NaOH) with the addition of 2.5 mM Na^+^ pyruvate, 100 units/ml penicillin, and 100 μg/ml streptomycin (20). A day after their isolation, the oocytes were injected with the relevant cRNAs: 5-HT_1A_ (1000 pg), and GIRK1 and GIRK2 (200 pg each), Gαi3 (1000 pg). 5-HT was purchased from Abcam. All other chemicals were purchased from Sigma Israel.

### Current measurements

The currents were measured 3 to 5 days after cRNA injection and recorded using two-electrode voltage-clamp amplifier (Warner OC 725C amplifier; Warner Instruments). The oocyte was placed in the recording bath containing ND96 solution and impaled with two electrodes pulled from 1.5 mm borosilicate capillaries (Warner Instruments). Both electrodes were filled with 3 M KCl solution. The electrode resistances were between 0.5 and 2 MΩ. 5-HT_1A_ receptor–mediated GIRK currents were measured in a 24 mM K^+^ solution (72 mM NaCl, 24 mM KCl, 1 mM CaCl_2_, 1 mM MgCl_2_, 5 mM Hepes, pH adjusted to 7.5 with KOH). In the Na^+^-free solution, the 72 mM NaCl was replaced by 72 mM *N*-methyl-d-glucamine, and the pH was adjusted with HCl. pCLAMP10 software (Axon Instruments) was used for data acquisition and analysis.

### Data analysis

The DR curves were fitted by the following equation:

Y = bottom + (X^Hill slope^) ∗ (top–bottom)/(X^Hill slope^ + EC50^Hill slope^), where Y is the normalized response, X is the concentration of 5-HT, Hill slope is the slope factor, and EC_50_ is the 5-HT concentration that gives the half-maximal response.

### Statistical evaluation

Statistical analysis was conducted using GraphPad Prism software (GraphPad Software, Inc). Significance was evaluated by Student’s two-tailed *t* test. Estimating the difference between the EC_50_ values was conducted by the extra-sum-of-squares *F* test.

## Data availability

All data associated with this work are contained within the article and supporting information.

## Supporting information

This article contains supporting information.

## Conflict of interest

The authors declare that they have no conflicts of interest with the contents of this article.

## Supporting information

Supporting Figures revision 16.4.Docx
