# Supplementary material for: The activity of the serotonergic 5-HT1A receptor is modulated by voltage and sodium levels
Source: J Biol Chem. 2022 Apr 22;298(6):101978. doi: 10.1016/j.jbc.2022.101978 (PMC9136116; doi:10.1016/j.jbc.2022.101978)
Supplement: Supporting Figures revision 16.4.Docx [file mmc1.docx]

**The** **activity of the serotonergic 5-HT_1A_ receptor is modulated by voltage and sodium levels**

Merav Tauber and Yair Ben Chaim^*^

Supporting information

**Figure S1.** Effect of 5-HT on GIRK channels. **A**. A representative recording from an oocyte expressing the GIRK channel. Application of 5 µM 5-HT did not evoke GIRK currents or inhibited basal GIRK currents. **B**. I-V curve of I_K_ before (black) and after (red) the application of 5-HT. The oocytes were voltage clamped to -100 mV and the currents after depolarizing pulses to various holding potentials from –100 mV to +40 mV at 10 mV increments were measured. Data is mean ± SD from 7 oocytes. The difference between the two conditions is not significant (paired t-test, *p>*0.2 for all voltages).

**Figure S2.** DR curves obtained from oocytes where recordings were conducted from the same oocyte at the two holding potentials (solid symbols and lines). Each point represents the mean (±SEM) from 7-29 oocytes. The EC_50_ values obtained for the two graphs (2.9 nM at -80 mV and 83.4 nM at +40 mV) were significantly different (*p<0.0001*). The DR curves constructed from data from all oocytes are shown for comparison (empty symbols and dashed lines; taken from Fig. 1C).

**Figure S3**. Buspirone and tandospirone are partial 5-HT_1A_ agonists. Each point represents the ratio between maximal GIRK current evoked by buspirone (**A**) or tandospirone (**B**) and the maximal current evoked by 5-HT in the same oocyte. The mean ± SE is shown as horizontal line. The efficacy of both agonists was not affected by the membrane potential (*p=*0.97 and 0.28 for **A** and **B**, respectively).
